# Human Salivary Histatin-1-Functionalized Gelatin Methacrylate Hydrogels Promote the Regeneration of Cartilage and Subchondral Bone in Temporomandibular Joints

**DOI:** 10.3390/ph14050484

**Published:** 2021-05-19

**Authors:** Changjing Shi, Yu Yao, Lei Wang, Ping Sun, Jianying Feng, Gang Wu

**Affiliations:** 1 School of Stomatology, Zhejiang Chinese Medical University, Hangzhou 310053, China; changj_shi@163.com (C.S.); yaoyu317@163.com (Y.Y.); 2 Wenzhou Institute, University of Chinese Academy of Sciences, Wenzhou 325000, China; wanglei20111213@163.com; 3 Department of Oral and Maxillofacial Surgery/Pathology, Amsterdam UMC and Academic Center for Dentistry Amsterdam (ACTA), Vrije Universiteit Amsterdam (VU), Amsterdam Movement Science, 1081 LA Amsterdam, The Netherlands; 4 Department of Oral Implantology and Prosthetic Dentistry, Academic Centre for Dentistry Amsterdam (ACTA), University of Amsterdam (UvA) and Vrije Universiteit Amsterdam (VU), 1081 LA Amsterdam, The Netherlands; 5 Key Laboratory of Oral Biomedical Research of Zhejiang Province, The Affiliated Hospital of Stomatology School of Stomatology, Zhejiang University School of Medicine, Hangzhou 310006, China; doctorsp@163.com

**Keywords:** histatin-1, Gel-MA hydrogels, cartilage repair, tissue engineering, temporomandibular joint

## Abstract

The avascular structure and lack of regenerative cells make the repair of osteochondral defects in the temporomandibular joint (TMJ) highly challenging in the clinic. To provide a viable treatment option, we developed a methacrylated gelatin (Gel-MA) hydrogel functionalized with human salivary histatin-1 (Hst1). Gel-MA is highly biocompatible, biodegradable, and cost-effective. Hst1 is capable of activating a series of cell activities, such as adhesion, migration, differentiation, and angiogenesis. To evaluate the efficacy of Hst1/Gel-MA, critical-size osteochondral defects (3 mm in diameter and 3 mm in depth) of TMJ in New Zealand white rabbits were surgically created and randomly assigned to one of the three treatment groups: (1) control (no filling material); (2) Gel-MA hydrogel; (3) Hst1/Gel-MA hydrogel. Samples were retrieved 1, 2, and 4 weeks post-surgery and subjected to gross examination and a series of histomorphometric and immunological analyses. In comparison with the control and Gel-MA alone groups, Hst1/Gel-MA hydrogel was associated with significantly higher International Cartilage Repair Society score, modified O’Driscoll score, area percentages of newly formed bone, cartilage, collagen fiber, and glycosaminoglycan, and expression of collagen II and aggrecan. In conclusion, Hst1/Gel-MA hydrogels significantly enhance bone and cartilage regeneration, thus bearing promising application potential for repairing osteochondral defects.

## 1. Introduction

Osteochondral defects in temporomandibular joints (TMJs) can result from acute injury, overloading, or abnormal immune response [1,2]. Osteochondral defects may lead to a lifetime of pain and restricted jaw motion for patients, even in daily activities such as talking, eating, and yawning [3].

In TMJs, the repair of osteochondral defects is highly challenging due to their limited self-regenerative potential [1]. Firstly, the avascular property of condyle cartilage tissue leads to the lack of a classic healing cascade, such as coagulation, inflammation, blood invasion, and accumulation of pluripotent mesenchymal stem cells (MSCs) [4]. Secondly, the chondrocytes in the surrounding cartilage tissue show poor migration and proliferation [4,5]. All these properties result in nearly no healing of TMJ cartilage. Thirdly, when defects further enlarge to affect subchondral bone tissues, the blood supply from bone tissues may, to some extent, trigger the classic healing pattern and enhance MSC migration [6]. However, such a blood supply and migration of MSCs are too limited to facilitate the complete repair of osteochondral defects [7]. Meanwhile, the reduced TMJ area will result in mechanical overloading on the remaining TMJ tissue, which may cause secondary mechanical damage to the TMJ [1]. In the clinic, osteochondral defects are managed mainly using various autografts, such as autologous chondrocyte implantation [8] and mosaicplasty [9,10]. These treatments show beneficial effects in the healing of osteochondral defects by providing chondrocytes. However, their usage is highly limited due to the limited availability of autografts and donor-site pain and morbidity [11]. Consequently, continuous efforts have been attempted to repair osteochondral defects.

Cartilage tissue engineering (TE) is a promising technique that elaborately involves various combinations of biomaterial scaffolds, bioactive agents, and stem cells to facilitate tissue reconstruction [12]. Scaffolds are an essential part of TE, for they provide a scaffolding matrix for cell migration and neo-tissue generation in the repair site. One of the most commonly used TE scaffolds is methacrylated gelatin (Gel-MA) that is a hydrolyzed form of and has the same chemical composition as collagen, thus bearing good biocompatibility without the risk of pathogen transmission as with collagen [13]. Gelatin contains many adhesive ligands, such as arginine–glycine–aspartic acid sequences so as to promote cell adhesion and migration [14]. Its excellent fluidity before crosslinking enables Gel-MA to flexibly fit into the complicated forms of defects [15]. Right after a short photo-crosslinking time, Gel-MA can transit from liquid to hydrogel and reach 50–60 kPa stiffness, which is favorable for cartilage and bone tissue formation [16]. Albeit so, Gel-MA still lacks intrinsic capacities of inducing angiogenesis and MSC homing, thus being unable to facilitate sufficient repair of osteochondral defects [17]. This limitation of Gel-MA may be approached by encapsulating MSCs [18] or bioactive agents [19]. In contrast to MSC-based TE, bioactive agent-based TE technique bears a series of advantages, such as low cost, wide sources, and low regulatory barriers in clinical translation [20,21]. An ideal bioactive agent should be able to induce both angiogenesis and MSC migration from the bone defect area to cartilage lesion site so as to facilitate the repair of osteochondral defects.

One of such bioactive agents is histatin-1 (Hst1) that belongs to a cationic and his-tidine-rich peptide family originally found in the saliva of higher primates [22]. Hst1 bears a potent capacity to stimulate the adhesion and migration of epithelial cells [23,24,25,26], fibroblasts [27], and osteoblasts [28,29]. Meanwhile, it can promote cell metabolic activity [26] and maintains cell viability in various adverse conditions [28,29]. Furthermore, Hst1 shows very strong angiogenetic properties [30]. Recently, in an in vivo ectopic bone induction model, we show that Hst1 significantly promotes bone morphogenetic protein 2 (BMP2)-induced angiogenesis and osteogenesis [31]. However, the effect of Hst1 on the repair of osteochondral defects remains unexplored. In this study, we hypothesized that Hst1-functionalized Gel-MA hydrogels could sufficiently promote the repair of critical-size osteochondral defects in TMJ.

## 2. Results

### 2.1. Postoperative Course

All animals recovered well postoperatively and had adequate food intake to maintain baseline body weight. There were no significant postoperative complications within the 4-week monitoring span.

### 2.2. Selection of Hst1 Dosage

**Figure 1:**
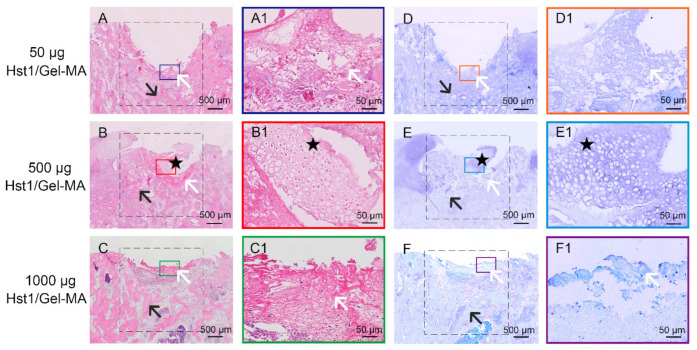
Light micrographs of H&E-stained (**A**–**C**) and toluidine blue-stained (**D**–**F**) tissue sections of rabbit condyles with critical-size (3 mm in diameter and 3 mm in depth) osteochondral defects that were treated using Gel-MA with 3 different doses of Hst1 (**A**,**D**) 50, (**B**,**E**) 500, and (**C**,**F**) 1000 μg per defect. The tissues were retrieved at 2 weeks postoperation and then subjected to histologic processing and sectioning. The dotted square area indicates the original defect area. Black star: immature cartilage cells; black arrow: newly formed subchondral bone; white arrow: Hst1/Gel-MA materials. Scale bar = 500 μm in **A**–**F**; Scale bar = 50 μm in **A1**–**F1**.

To determine the optimal dosage of Hst1 to promote osteochondral repair, a preliminary study was carried out to explore the repair effects of Gel-MA with 3 different doses of Hst1: 50, 500, and 1000 μg per defect. At 2 weeks, only mild new bone formation but not cartilage formation was detected in the defects with 50 μg Hst1/Gel-MA (Figure 1A–A1, D–D1). In contrast, much more new bone formation and several islands of new cartilage could be observed in the defects treated with 500 μg Hst1/Gel-MA (Figure 1B–B1, E–E1). Interestingly, the defects treated with 1000 μg Hst1/Gel-MA were almost fulfilled with a tremendous amount of new bone tissue (Figure 1C–C1, F–F1), while newly formed cartilage tissue was rarely detected. Thus, we chose 500 μg Hst1 per defect in the following experiments.

### 2.3. Macroscopic Evaluation

**Figure 2:**
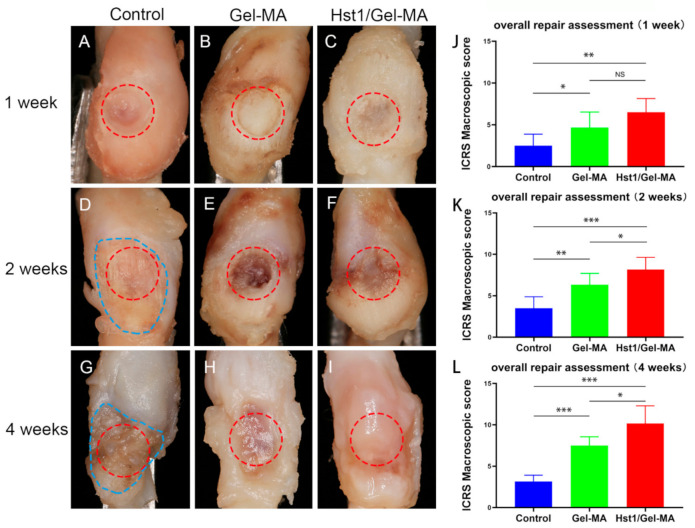
Photographs and macroscopic evaluation of rabbit condyle osteochondral defects of TMJ, which were treated as control group (**A**,**D**,**G**), Gel-MA group (**B**,**E**,**H**) and Hst1/Gel-MA group (**C**,**F**,**I**). The tissues were retrieved at 1, 2, and 4 weeks post operation and then photographed and scored using ICRS macroscopic scores (**J**–**L**). The red circle area represents the original defect area, and the blue irregular area represents the enlarged absorption area (*n* = 6, * *p* < 0.05; ** *p* < 0.01; *** *p* < 0.001 and NS = not significant).

At 1 week, the defects were still hollow and showed distinct edges from the surrounding cartilage in the control group (Figure 2A). In Gel-MA group, the defects were filled with white rough tissue with a clear border. The surface of the defects remained concave (Figure 2B). Moreover, the defects of Hst1/Gel-MA group were filled with pale pinkish tissue. There was little depression in the defects and the margin with normal cartilage was indistinct (Figure 2C). The International Cartilage Repair Society (ICRS) macroscopic scores of Hst1/Gel-MA group were significantly higher than the scores of Gel-MA group and control group (*p* < 0.05) (Figure 2J).

At 2 weeks, the tissues in the vicinity of the original defect further collapsed, resulting in pronounced extension of the defect order and the enlargement of the defect area (Figure 2D). The defect surfaces of Gel-MA group showed a red, irregular, depressed morphology without visible collapse of the surrounding tissues (Figure 2E). The defect border was still sharply defined. In comparison, cartilage defects were filled with pale red tissue with obscure demarcation from surrounding cartilage in Hst1/Gel-MA group (Figure 2F). The ICRS macroscopic scores of Hst1/Gel-MA group were significantly higher than those of Gel-MA group and control group. Statistically significant differences could be found between the two groups at this time point (*p* < 0.05) (Figure 2K).

At 4 weeks, further enlargement of the defect border could be observed in the control group. Irregular fibrous-like tissue formation was found in the defects (Figure 2G). In comparison, the defects of Gel-MA group were filled with reddish tissue, and the surface remained depressed (Figure 2H). In contrast, in the Hst1/Gel-MA group, there was firm, smooth, cartilage-like tissue filled in the defects. Additionally, the color and morphology of the newly regenerated tissue were similar to the adjacent normal cartilage (Figure 2I). The ICRS macroscopic scores of Hst1/Gel-MA group were significantly higher than those of the Gel-MA group (*p* < 0.05) and control group (*p* < 0.001). Notably, the mean score of Hst1/Gel-MA group was about three times that of the control group (Figure 2L).

### 2.4. Histologic Observation and Histomorphometric Analysis on HE-Stained Tissue Sections

**Figure 3:**
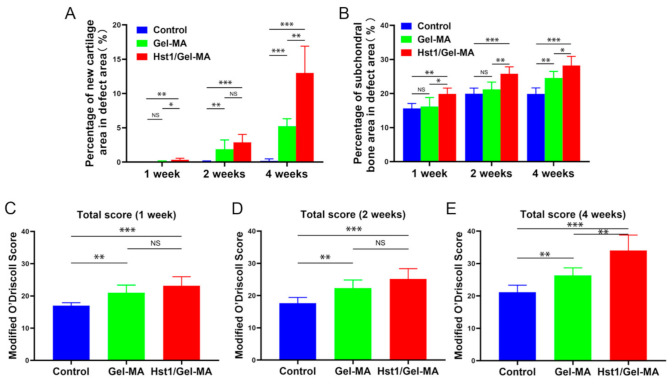
Quantitative analysis of newly formed cartilage (**A**) and subchondral bone area (**B**), and the MODS evaluation for repaired osteochondral defects at 1, 2, and 4 weeks postimplantation (**C**–**E**). (*n* = 6, * *p* < 0.05, ** *p* < 0.01, *** *p* < 0.001 and NS = not significant).

At 1 week, in the control group, nearly no newly regenerated tissue could be detected in the defects of the control group with the bone surface uncovered. There was still a large amount of porous Gel-MA material in both the Gel-MA and Hst1/Gel-MA groups. However, only in the Hst1/Gel-MA group, some immature chondrocytes and other cells infiltrated into the porous structures of Gel-MA. The area percentages of the newly formed subchondral bone tissue and cartilage in the Hst1/Gel-MA group were significantly higher than Gel-MA group (*p* < 0.05) and control group (*p* < 0.01). However, there were no significant differences between the Gel-MA and control groups (Figure 3A,B). Meanwhile, the modified O’Driscoll score (MODS) in the groups of Hst1/Gel-MA and Gel-MA were significantly higher than that MODS in the control group, whereas no significant difference in MODS could be detected between Hst1/Gel-MA group and Gel-MA group (Figure 3C).

At 2 weeks, the defects in the control group still showed absence of tissue repair with the subchondral bone surface still exposed. In the Gel-MA group, few cells infiltrated into the porous Gel-MA material. In comparison, new cartilage and bone tissue formed and replaced the Gel-MA material gradually in Hst1/Gel-MA group. The area percentages of the newly formed cartilage in the Hst1/Gel-MA and Gel-MA groups were significantly higher than for the control group (*p* < 0.01) (Figure 3A), whereas no significant difference in the area percentages of the newly formed cartilage could be detected between the Hst1/Gel-MA and Gel-MA groups (Figure 3A). Meanwhile, the area percentages of the newly formed subchondral bone tissue in the Hst1/Gel-MA group were significantly higher than Gel-MA group (*p* < 0.01) and control group (*p* < 0.001) (Figure 3B), whereas there were no significant difference between the Gel-MA and control groups (Figure 3B). In addition, the Hst1/Gel-MA and Gel-MA groups had significantly higher MODS than the control group (*p* < 0.01) (Figure 3D). However, no significant difference in MODS could be detected between the Hst1/Gel-MA and Gel-MA groups (Figure 3D).

**Figure 4:**
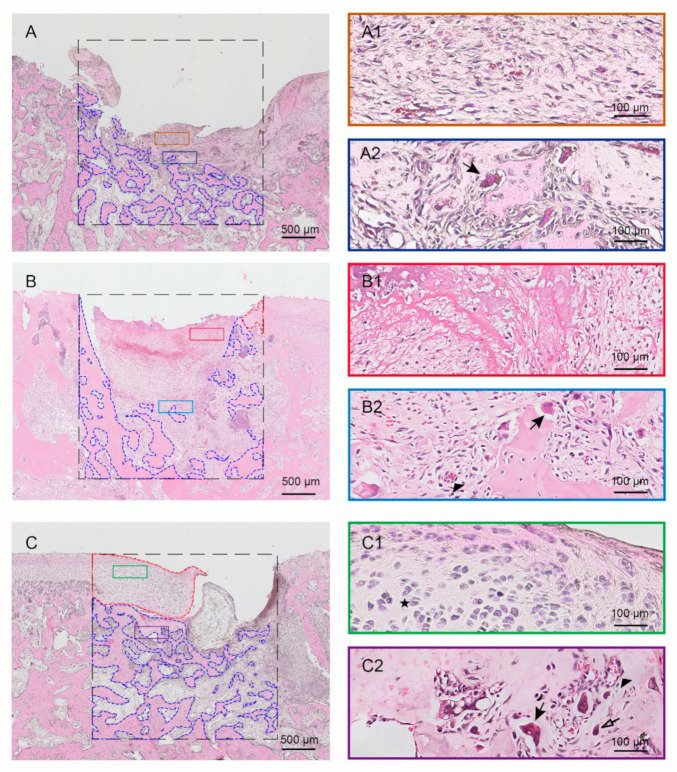
Light micrographs of H&E-stained tissue sections of rabbit condyles with critical-size (3 mm in diameter and 3 mm in depth) osteochondral defects that were treated as control group (**A**,**A1**,**A2**), Gel-MA group (**B**,**B1**,**B2**) and Hst1/Gel-MA group (**C**,**C1**,**C2**). The tissues were retrieved at 4 weeks and then subjected to histologic processing and sectioning. The dotted square area is the original defect area (3 mm × 3 mm). The newly formed cartilage is delineated by the red dotted line, and the new subchondral bone is delineated by the blue dotted line. Solid arrow: osteoclasts; hollow arrow: osteoblasts; black star: immature cartilage cells; triangle: neovascularization. Scale bar = 500 μm in **A**–**C**; Scale bar = 50 μm in **A1**–**C2**.

At 4 weeks, the defect area in control group was significantly enlarged and remained hollow with a layer of fibrous tissue on its surface (Figure 4A–A1). In the Gel-MA group, only a few cells could be detected within the remaining Gel-MA material. There was mainly fibrous tissue along with Gel-MA material in the defects (Figure 4B–B1). Meanwhile, the new subchondral bone formation could be detected (Figure 4B–B2). In comparison, large areas of newly formed cartilage were detected in the Hst1/Gel-MA group (Figure 4C). Notably, chondrocytes exhibited a typical lacunae structure with columnar alignment (Figure 4C1). Moreover, right above the newly formed chondrocyte layer, there appeared to be a fibrous layer—similar to that found in the native mandibular condylar cartilage (Figure 4C1). At the bottom of the defect, improved subchondral bone remodeling with a large number of infiltrated osteoblasts and osteoclasts was observed (Figure 4C2). Evidence of neovascularization was detected in the junction between subchondral bone and hydrogel (Figure 4C2). Additionally, the mean area percentage of newly formed cartilage in Hst1/Gel-MA group was almost two times that of the Gel-MA group (*p* < 0.01), and they were both significantly higher than that of the control group (*p* < 0.001) (Figure 3A). The area percentages of the newly formed subchondral bone tissue in Hst1/Gel-MA group were significantly higher than for the Gel-MA group (*p* < 0.05) and control group (*p* < 0.001) (Figure 3B). Meanwhile, the Hst1/Gel-MA group had significantly higher MODS compared to the Gel-MA group (*p* < 0.01) and control group (*p* < 0.001). Gel-MA group had significantly higher MODS than the control group (*p* < 0.01) (Figure 3E). All the light micrographs of the HE-stained tissue sections for all samples at 4 weeks for this quantification are available in Figure S1.

### 2.5. Histologic Observation and Histomorphometric Analysis on Sections with Alcian Blue Staining or Masson’s Trichrome Staining

**Figure 5:**
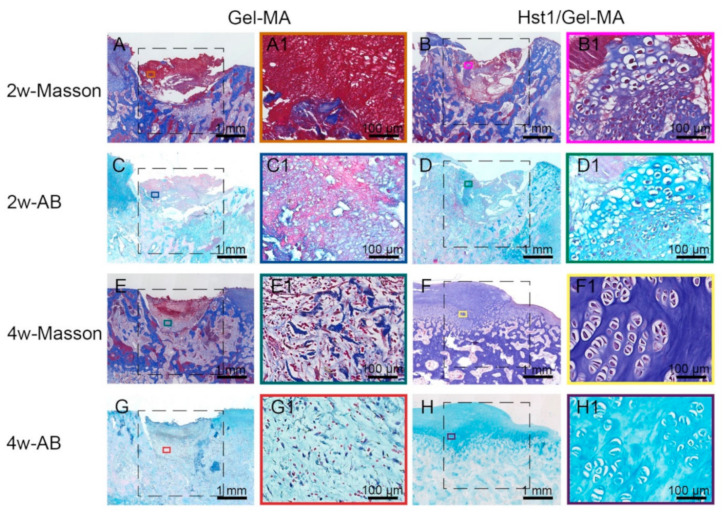
Light micrographs of Alcian blue-stained and Masson’s trichrome-stained tissue sections of repaired osteochondral defects in the Gel-MA and Hst1/Gel-MA groups. Masson’s trichrome staining at 2 weeks (**A**,**B**) and 4 weeks (**E**,**F**); Alcian blue staining at 2 weeks (**C**,**D**) and 4 weeks (**G**,**H**). The dotted square area indicates the original defect area (3 mm × 3 mm). Scale bar = 1 mm in **A**–**H**; Scale bar = 100 μm in **A1**–**H1**.

**Figure 6:**
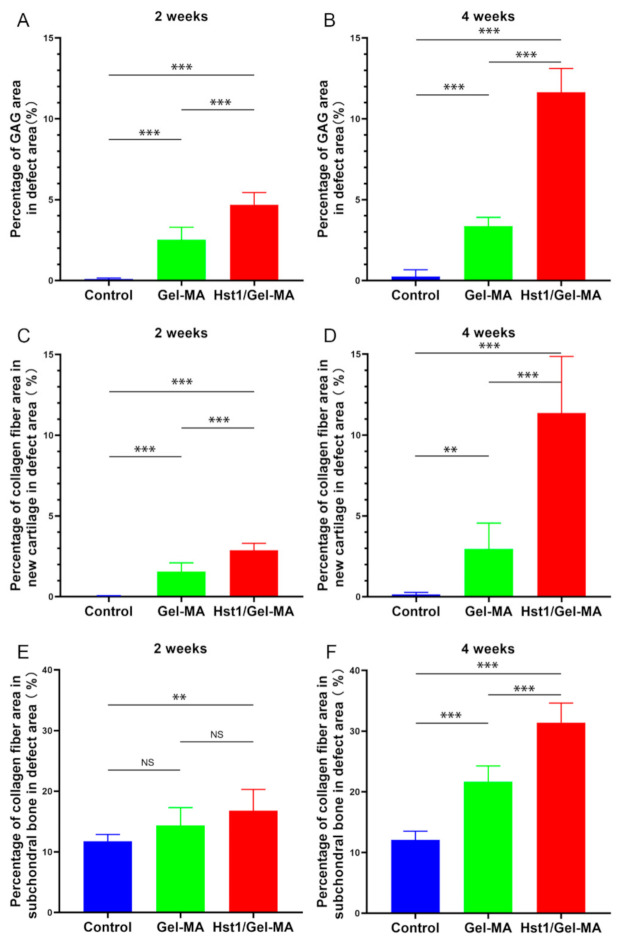
Quantitative analysis of the formation of GAG and collagen fiber in new cartilage and subchondral bone in the defect area at 2 and 4 weeks. Percentage of GAG area in defect area at 2 (**A**) and 4 weeks (**B**). Percentage of collagen fiber area in new cartilage in defect area at 2 (**C**) and 4 weeks (**D**). Percentage of collagen fiber area in subchondral bone in defect area at 2 (**E**) and 4 weeks (**F**) (*n* = 6, ** *p* < 0.01, *** *p* < 0.001 and NS = not significant).

At 2 weeks, Masson’s trichrome staining exhibited very little blue staining in the Gel-MA materials, which revealed that nearly no collagen fibers especially collagen type II formation (Figure 5A–A1). Alcian blue staining also showed little to no glycosaminoglycan (GAG) production with staining almost absent in the defects (Figure 5C–C1). In comparison, the existence of collagen fibers, especially collagen type II, in the newly formed cartilage was demonstrated by Masson’s trichrome staining in the Hst1/Gel-MA group (Figure 5B–B1). The Alcian blue staining also revealed the GAG content of the extracellular matrices (ECM) in the cartilage islands in the defects (Figure 5D–D1). Histomorphometric analysis revealed that the Hst1/Gel-MA group had significantly higher GAG and collagen fiber content than the Gel-MA and control groups (*p* < 0.001) (Figure 6A,C,E).

At 4 weeks, in the Gel-MA group, little collagen deposition, as stained blue by Masson’s trichrome, was found in the Gel-MA material (Figure 5E–E1). Alcian blue staining exhibited rare GAG formation in the defects (Figure 5G–G1). In the Hst1/Gel-MA group, Masson’s trichrome staining showed strongly blue staining in the newly formed cartilage, indicating collagen fibers were produced in the ECM in the repair tissues (Figure 5F–F1). Meanwhile, Alcian blue staining confirmed the presence of GAG in the ECM in the newly formed cartilage (Figure 5H–H1). Histomorphometric analysis showed Hst1/Gel-MA group had significantly higher GAG and collagen fiber content than the Gel-MA and control groups (*p* < 0.001) (Figure 6B,D,F).

### 2.6. Immunohistochemical Evaluation

**Figure 7:**
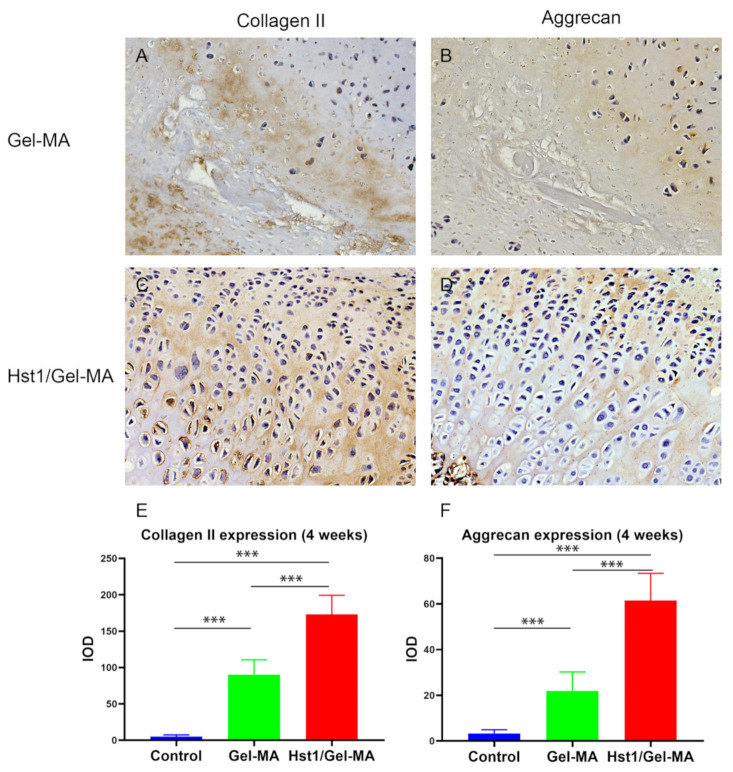
Light micrographs of immunohistochemically stained tissue sections of collagen II (**A**,**C**) and aggrecan (**B**,**D**) in the Gel-MA group (**A**,**B**) and Hst1/Gel-MA group (**C**,**D**). The tissues were retrieved at 4 weeks postoperation and then subjected to immunohistochemical processing and sectioning. Quantitative analysis of collagen II (**E**) and aggrecan (**F**) expression at 4 weeks. Scale bar = 100 μm in **A**–**D**. (*n* = 6, *** *p* < 0.001).

In the Hst1/Gel-MA group, the expression of collagen II and aggrecan increased gradually over time, approaching that of normal cartilage by 4 weeks after implantation (Figure 7C,D). However, in Gel-MA group, there was almost no presentation of collagen II and aggrecan in the repaired tissue at 1 and 2 weeks, while slight staining of collagen II and aggrecan is visible in the Gel-MA group at 4 weeks (Figure 7A,B). No positive immunostaining for collagen II and aggrecan was observed in the control group at any time point. The Hst1/Gel-MA group has significantly higher collagen II and aggrecan expression compared to the Gel-MA and control groups at 4 weeks (*p* < 0.001) (Figure 7E,F).

## 3. Discussion

The main difficulty in the repair of critical-size osteochondral defects in TMJs mainly lies in insufficient self-regenerative cells in lesion areas [32]. Most of the current clinical therapies try to approach this problem by auto-transplanting chondrocytes or cartilage, which is restricted by their very limited availability and donor site morbidity [11]. In this study, to provide a viable treatment option, we developed a Hst1-functionalized Gel-MA hydrogel to combine the chondroconductive properties of Gel-MA and the potent angiogenetic and cell-activating capacity of Hst1. To our best knowledge, this was the first study to show the effects of Hst1 on the repair of osteochondral defects in TMJ. Our data show that Hst1/Gel-MA hydrogel group possessed a significant higher ICRS score and MODS in comparison with the Gel-MA group and control group. Furthermore, histomorphometric analysis showed significantly higher expression of collagen II, aggrecan, collagen fiber, GAG, and more newly formed subchondral bone and cartilage in Hst1/Gel-MA hydrogel group than the Gel-MA group and control group. Our data suggest a promising application potential of Hst1/Gel-MA hydrogels in promoting the repair of critical-size osteochondral defects in TMJ.

The spontaneous healing efficacy of osteochondral defects in TMJ is highly dependent on the size, shape, and depth of the lesions. For example, nearly no spontaneous tissue repair can be detected in a defect 2 mm diameter and 2 mm deep in rabbit TMJ 3 weeks postoperatively, since the lesions are largely restricted in cartilage or on the bone–cartilage interface that bears limited resources of blood supply and regenerative cells [33]. When defects are deeper and affect the subchondral bone area, the blood supply from the subchondral bone area will trigger a classical healing cascade and deliver self-regenerative MSCs [6]. In this situation, the healing efficacy shows a diameter-dependent pattern. In deep (3 mm in-depth) and non-critical-size (1 mm in diameter) defects, the healing efficacy varied with some defects being filled with disorganized fibrocartilage and others filled with a nearly continuous layer of cartilage at 6 weeks postoperation [3]. Most in vivo studies reported that when defects had a diameter of less than 3 mm, they may partially heal [34,35]. Therefore, the osteochondral defects with a 3 mm depth and 3 mm diameter in rabbit TMJs can be considered as critical-size defects. In our current study, we surgically created critical-size osteochondral defects (3 mm in depth and 3 mm in diameter) in the TMJ of rabbits to investigate the healing efficacy of Hst1/Gel-MA hydrogel. We found that there was only some bone formation but nearly no newly formed cartilage in the untreated defects. This finding suggests that blooding and blood-borne MSCs from subchondral bone were insufficient in triggering chondrogenesis. Instead, the defects further collapsed and caused breakdown of the surrounding tissues. This phenomenon might be attributed to the secondary mechanical damage and further breakdown surrounding the lesions [1] since the TMJ load may be redistributed to the TMJ condyle with a much lower surface area.

As alternative to auto-transplantation in the clinic, a large variety of TE techniques have been developed to repair osteochondral defects. Scaffold material is an indispensable element for TE to facilitate cell migration, proliferation, and differentiation as well as the slow release of bioactive agents [15]. In recent TE techniques, there is a trend in the design of material scaffolds to contain three parts with different physicochemical and biological properties in order to facilitate the regeneration of bone, cartilage, and the osteochondral interface separately [36]. However, such complicated designs are less favorable for their industrial fabrication and clinical application. To develop a filling material with more promise for clinical application, we adopted Gel-MA hydrogels as the scaffold material. Gel-MA hydrogels possess a series of advantages, such as in being biocompatible, biodegradable, nonimmunogenic, physicochemical modifiable, and cost-effective [37]. They are highly similar to the natural ECM both in provide a free entrance for nutrients and in supporting cellular growth [38]. Furthermore, Gel-MA can also support its encapsulated chondrocytes to produce the ECM such as through proteoglycan and type II collagen deposition as well as chondrogenesis-related gene expression [39]. In our study, compared with the absence of tissue repair in the defects and even further breakdown of the surrounding tissues in control group, cell infiltration in the non-degraded scaffold of the Gel-MA group could be detected with subchondral bone deposition at 4 weeks. In addition, Masson’s trichrome staining and Alcian blue staining confirmed that there were few collagen fibers and little GAG formation in the defects, which indicated that Gel-MA supported cartilage ECM formation. Meanwhile, the score of ICRS and MODS of Gel-MA group were significantly higher than the control group (*p* < 0.05). However, Gel-MA hydrogel alone is not sufficient to heal the critical-size osteochondral defects in TMJ.

To further promote the healing efficacy, scaffold materials need to be functionalized by bioactive agents, particularly proteinous growth factors such as BMP2 and transforming growth factor-β (TGF-β). Osteochondral regeneration is delicately regulated by several macromolecular protein growth factors, such as bone morphogenetic proteins (BMPs). BMP2 belongs to BMP family, a group of proteinaceous growth factors under the TGF-β superfamily [40]. The classical role for BMP2 is considered to be in the induction of (ectopic) cartilage and bone formation [41,42]. In the USA, a product containing recombinant human (rh)BMP2 in absorbable collagen has already been approved for clinical application in nonunion bone fractures and spinal fusions [43]. However, the use of BMP2 is associated with the concern that BMP2 induces chondrocyte hypertrophy followed by cartilage calcification [44], which compromises the regeneration of cartilage layer. In the field of TE, there is a trend to combine growth factors to specifically induce and maintain the zonal phenotypes of cartilage. For example, cultivation of MSCs with TGF-β1 (3 ng/mL) and BMP-7 (300 ng/mL) induced the synthesis of superficial zone protein, a marker for chondrocytes in the superficial zone [45], whereas insulin-like growth factor-1 (IGF-1) did not [46,47]. Cultivation of chondrocytes isolated from the middle zone with TGF-β1 (30 ng/mL) and IGF-1 (100 ng/mL) significantly increased collagen synthesis [48,49]. TGF-β1 also contributes to the maintenance of calcified cartilage zone as deletion of the TGF-β1 receptor gene from chondrocytes delayed endochondral ossification [50] and cultivation of bovine hypertrophic chondrocytes with a combination of TGF-β1 (30 ng/mL) and 3% (*w/v*) hydroxyapatite (HA) increased matrix deposition and mineralization [50,51]. However, such complex zonal arrangement of growth factors is less feasible for biomedical application. In addition, proteinous growth factors bear low production yield and, thus, high cost, which further limits their clinical application. As promising alternatives to the macromolecular protein growth factors, peptides can be chemically and standardly synthesized, thus bearing better reproducibility and yielding efficiency [52].

In this study, we adopted Hst1—a salary bioactive peptide that can promote a series of cell activities, such as cell adhesion [23,24,27,30,53], spreading [23,24], migration [22], cell–cell adhesion [24,54], angiogenesis [30], and metabolic activity [26,28,29,55]. Furthermore, Hst1′s cell-activating effects seem to be independent of cell types, thus bearing broad applicability. Such a property is of paramount importance particularly for the application in repairing osteochondral defects, which requires promotion of the functions of both osteoblast and chondrocytes [43,44]. To identify the optimal dosage of Hst1, we first performed a preliminary study to investigate the dose-dependent effects of Hst1 on the repair of critical-size osteochondral defects. We found that 50 μg Hst1/Gel-MA was associated with insufficient new bone and cartilage formation, while 1000 μg Hst1/Gel-MA resulted in overstimulation of bone regeneration with compromised cartilage formation. It seems that over-dosed Hst1 will be more beneficial for bone regeneration and detrimental for cartilage formation. This may be due to the angiogenetic effect of Hst1. As for other cell types, Hst1 can promote the adhesion, spreading, and migration of endothelial cells [30]. Furthermore, Hst1 can also promote vascular morphogenesis and angiogenesis through activating Rac1 via a RIN2/Rab5/Rac1 signaling pathway axis [30]. Moreover, it is well established that overstimulated angiogenesis can cause cartilage resorption and bone deposition [56]. Consequently, overdosed Hst1 may harm cartilage regeneration through overstimulated angiogenesis.

On the other hand, angiogenesis is an indispensable biological event to deliver nutrients, oxygen, and hematopoietic stem cells to cartilage defects, thereby facilitating cartilage regeneration. Consequently, elaborate modulation of Hst1 dosage is critical for balanced bone and cartilage formation. In this study, we showed that 500 μg Hst1/Gel-MA hydrogels significantly promoted the deposition of ECM compositions (collagen II and aggrecan) and the formation of zonally structured cartilage in the defects in comparison with Gel-MA hydrogels and the control (*p* < 0.05). The score of ICRS and MODS in Hst1/Gel-MA group were also significantly higher than in the Gel-MA group. In addition, numerous chondroblasts in the newly formed cartilage layer and osteoblasts and osteoclasts in the subchondral bone could be observed in our study, indicating a high level of metabolism and remodeling. Consequently, 500 μg Hst1/Gel-MA hydrogel was proven to be the most efficacious in repairing osteochondral defects in the TMJ. In comparison with the complicated designed osteochondral constructs, the 500 μg Hst1/Gel-MA hydrogel is much more feasible and simpler, thus bearing more promising potential in biomedical application.

However, hitherto, there are no reports about how Hst1 facilitates chondrogenesis. One mechanism can be its well-established promoting effect on the adhesion, spreading, and migration of hematopoietic stem cells from subchondral bone and/or the chondrocytes from adjacent cartilage. The latter may be possible since that newly formed cartilage islands first occurred in the vicinity of the remaining cartilage. However, it is also well known that mature chondrocytes hardly migrate to repair cartilage defects [4]. Therefore, it may also be explained that Hst1 induced the secretion of growth factors and cytokines from mature chondrocytes so as to stimulate the chondrogenic differentiation of hematopoietic stem cells. It has recently been established that Hst1 promotes osteogenic differentiation [57,58]. Furthermore, our recent study shows that Hst1 significantly promotes BMP2-induced angiogenesis and osteogenesis [31]. Therefore, the interaction of exogenous Hst1 and endogenous BMP2 may be also responsible for the promotion of cartilage formation in the current study. Previous studies suggested that Hst’s effect is associated with numerous signaling pathways, such as RAC1 [30], ERK [23], p38 [57], and NF-ĸB [58], among others. However, there has been no report on the effect of Hst1 on chondrocytes.

## 4. Materials and Methods

### 4.1. The Preparation of Hydrogel Prepolymer Solution and Hst1

The freeze-dried Gel-MA was purchased from Wenzhou institute (Wenzhou, China). 200 mg of freeze-dried Gel-MA macromer was dissolved in 1 ml of PBS containing 0.5% (*w/v*) 2-hydroxy-1-(4-(hydroxyethoxy)phenyl)-2-methyl-1-propanone (Irgacure2959, CIBA Chemicals, Basel, Switzerland) at 80 °C and then filtered with a bacteria filter. The prepolymer solution was stored at 40 °C in a constant temperature water bath. It was prepared freshly before surgery and stored in bacteria-free bottles.

The lyophilized linear Hst1 peptide was obtained from the University of Amsterdam (Amsterdam, Holland) and stored at −20 °C. Hst1 peptide (500 μg) was dissolved in 21.1 μL (defect volume) prepolymer solution to prepare as the implantation.

### 4.2. Group Set-Up

In order to screen the optimal dosage of Hst1, we first performed a preliminary experiment, where we selected three doses of Hst1: 50, 500, and 1000 μg per defect according to the findings in our recent publication [31]. In that study, we evaluated the promoting effect of Hst1 at 50, 200, 500 μg/sample on BMP2-induced osteogenesis and angiogenesis. Micro-CT analysis showed that Hst1 dose-dependently increased the total volume of BMP2-induced newly formed bone. Furthermore, 50 μg of Hst1 per sample could already significantly enhance trabecular number and decrease trabecular separation of new bone. Immunostaining analysis showed that 500 μg of Hst1 per sample significantly enhanced levels of the BMP2-induced osteogenic markers (Runx2, Collagen) and angiogenic markers (FGF-2, CD105, and CD31). According to these results, in the preliminary experiment of the current study, we chose 50 and 500 μg, and also added 1000 μg to see whether we could get more beneficial effects with the higher dose. Two weeks postsurgery, animals were sacrificed and subjected to histological process. According to the histological staining result, 500 μg Hst1/Gel-MA was associated with a sufficient and balanced bone and cartilage formation in comparison with 50 μg Hst1/Gel-MA and 1000 μg Hst1/Gel-MA (see details in Section 2.2). Consequently, we chose 500 μg Hst1 per defect in the following experiments.

In the formal experiments, we set up 3 groups: control group (the defects receiving no filling material); Gel-MA alone group (the defects filled with Gel-MA hydrogels without Hst1); and 500 μg Hst1/Gel-MA group (the defects filled with 500 μg Hst1-functionalized Gel-MA hydrogels). Fifty-four adult male New Zealand white rabbits (Zhejiang Chinese Medical University, Hangzhou, China) were randomly divided into the 3 groups with 18 animals in each group. Six animals per group per time point were euthanized at 1, 2, and 4 weeks postsurgery.

### 4.3. Animal Surgery

**Figure 8:**
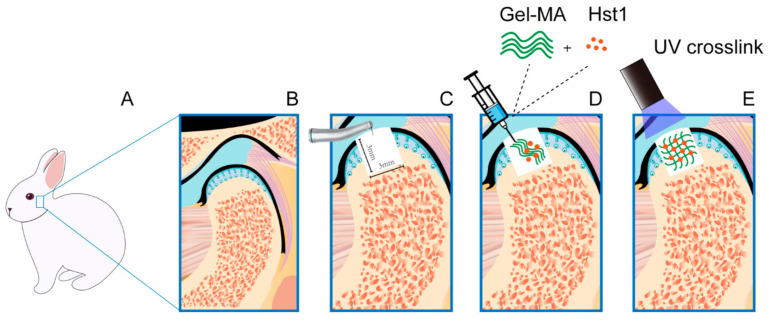
The schematic diagram (**A**–**E**) of the rabbit mandibular joint condyle critical-size (3 mm in diameter and 3 mm in depth) osteochondral defects model (3 mm × 3 mm) established procedure.

The animal study was reviewed and approved by the Institutional Animal Care and Use Committee of Zhejiang Chinese Medical University (no. IACUC-20180625-04). With the animals under general anesthesia, a preauricular skin incision was performed over the right TMJ. Then the condyle was exposed, and an osteochondral defect, 3 mm in diameter and 3 mm in depth, was created in the condyle with a 3 mm diameter drill. Surgery was performed on the right condyle of the TMJ in all 54 animals, and the left remained intact. In the control group, the defects were created but not treated. Gel-MA group was given Gel-MA hydrogels. Hst1-functionalized Gel-MA hydrogels were injected into the defect sites in Hst1/Gel-MA group. The Gel-MA prepolymer solution was photopolymerized by ultraviolet rays (365 nm, 90 s) in the Gel-MA and Hst1/Gel-MA groups (Figure 8). The articular capsule and skin were closed independently with nylon sutures. After all procedures were completed, animals were returned to the animal facility. Penicillin (1:100,000) was intraperitoneally administered every 24 h for the first three days after surgery.

### 4.4. Macroscopic Score Evaluation

The right TMJ and surrounding tissues were retrieved for macroscopic score evaluation. The gross appearance of the defect sites was photographed and blindly scored by 3 independent observers using ICRS macroscopic scoring system which contains four categories: the degree of repair, integration to border zone, macroscopic appearance, and overall repair assessment [59].

### 4.5. Histological Examination

The specimens for histological analysis were fixed in 10% neutral formalin (Sigma-Aldrich, St. Louis, MO, USA) after macroscopic evaluation for 24 h at 4 °C, and then decalcified with 10% ethylenediaminetetraacetate (EDTA)-buffered saline solution (Sigma-Aldrich, St. Louis, MO, USA) for 28 days. The specimens were cut into 4 μm thickness after embedded in paraffin (Sigma-Aldrich, St. Louis, MO, USA). The sagittal sections were stained with hematoxylin and eosin, Masson’s trichrome, and Alcian blue (Sigma-Aldrich, St. Louis, MO, USA). At the same time, the sections were immunohistochemically analyzed for type II collagen (COL II) and aggrecan content, with procedures performed following the manufacturer’s protocol. Under an Olympus CX51 microscope (Olympus Corporation, Tokyo, Japan), all slices were examined, and a digital charge-coupled device camera (QuantEM 512SC; Photometrics, Tucson, AZ, USA) was used for recording. The sections were blindly scored by 3 different experienced pathologists using MODS [60].

### 4.6. Histomorphometry Analysis

The original defect area is bounded by a 3 mm × 3 mm square in all slices of the samples. The top of the square is aligned with the cartilage surface on both sides. Percentage of newly formed subchondral bone area, new cartilage area, collagen fiber area, and GAG area in defect area was detected and calculated by Image Pro Plus software (v. 6.0.0.260; Media Cybernetics, Rockville, MD, USA). The areas of newly formed subchondral bone and new cartilage were selected through a manual delineation by researchers (as indicated by the blue (for bone tissue) and red (for cartilage tissue) dotted lines in Figure 4) but not the gray value-based automatic selection by software. The content of collagen II and aggrecan was expressed as integrated optical density (IOD) measured using ImagePro Plus 6.0.

### 4.7. Statistical Analysis

Statistical analysis between all groups was performed using one-way ANOVA and Tukey’s HSD post hoc test using the SPSS 18.0 statistical analytical software (SPSS Inc., Chicago, IL, USA). All the data are presented as mean ± standard deviation (SD). In all cases, *p*-values < 0.05 were considered to indicate a statistically significant difference, and *p*-values < 0.01 and 0.001 were considered highly significant differences.

## 5. Conclusions

In this study, we, for the first time, demonstrated that Hst1-functionalized Gel-MA hydrogel was a simple, feasible, and efficacious construct to facilitate the repair of osteochondral defects of TMJ in rabbits. In contrast to the current therapies, such as auto-transplantation of chondrocytes or cartilages, Hst1/Gel-MA bears unlimited availability and no donor-site morbidity. All these properties confer Hst1/Gel-MA with promising potential in biomedical application. Further studies should be performed to investigate the effects and molecular mechanisms of Hst1 on chondrogenesis.

## Data Availability

Data are contained within the article and associated Supplemental Materials.

## Supplementary Materials

The following are available online at https://www.mdpi.com/article/10.3390/ph14050484/s1, Figure S1. Light micrographs of H&E-stained tissue sections of rabbit condyles with critical-size (3 mm in diameter and 3 mm in depth) osteochondral defects that were treated as control group (A1-A6), Gel-MA group (B1-B6) and Hst1/Gel-MA group (C1-C6).
