# Supplementary material for: Human Salivary Histatin-1-Functionalized Gelatin Methacrylate Hydrogels Promote the Regeneration of Cartilage and Subchondral Bone in Temporomandibular Joints
Source: Pharmaceuticals (Basel). 2021 May 19;14(5):484. doi: 10.3390/ph14050484 (PMC8159088; doi:10.3390/ph14050484)
Supplement: Supplementary file 1 [file pharmaceuticals-14-00484-s001.zip › pharmaceuticals-1154415-SI.pdf]

Supporting Information

Human salivary histatin-1-functionalized Gelatin Methacrylate Hydrogels promotes the regeneration of cartilage and subchondral bone in temporomandibular joint

Changjing Shi [1†], Yu Yao [1†], Lei Wang[2,3,4†], Ping Sun[5], Jianying Feng [1,*] and Gang Wu[3,4*]

1 School of Stomatology, Zhejiang Chinese Medical University, Hangzhou Zhejiang 310053, China; 974876934@qq.com (C.J.S.); 3120374125@qq.com (Y.Y.)
2 Wenzhou Institute of Biomaterials & Engineering, University of Chinese Academy of Science, Wenzhou, Zhejiang 325027, China; wanglei20111213@163.com (L.W.)
3 Department of Oral and Maxillofacial Surgery/Pathology, Amsterdam UMC and Academic Center for Den-tistry Amsterdam (ACTA), Vrije Universiteit Amsterdam (VU), Amsterdam Movement Science, Amsterdam, The Netherlands;
4 Department of Oral Implantology and Prosthetic Dentistry, Academic Centre for Dentistry Amsterdam (ACTA), University of Amsterdam (UvA) and Vrije Universiteit Amsterdam (VU), 1081 LA Amsterdam, Netherlands;
5 The Affiliated Hospital of Stomatology, School of Stomatology, Zhejiang University School of Medicine, and Key Laboratory of Oral Biomedical Research of Zhejiang Province, Hangzhou, Zhejiang, 310006, China. doc-torsp@163.com (P.S.)

† The authors contributed equally
* Correspondence: twohorsejy@163.com; Tel.: +86 13093798116 (J.Y.F.); g.wu@acta.nl; Tel.: +31 20 5980866 (G.W.)

Figure S1

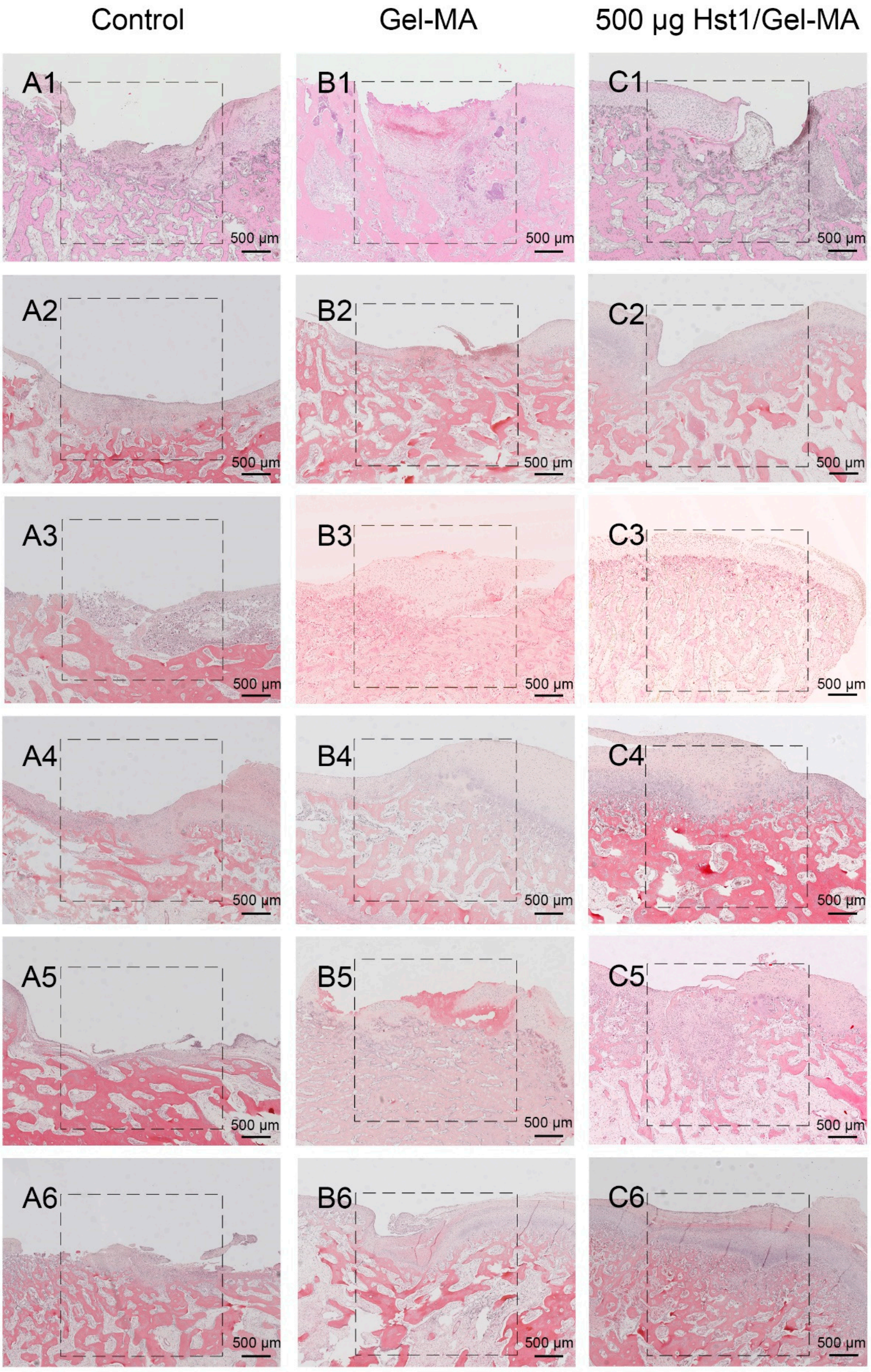

Figure S1. Light micrographs of H&E-stained tissue sections of rabbit condyles with critical-size (3 mm in diameter and 3 mm in depth) osteochondral defects that were treated as control group (A1–A6), Gel-MA group (B1–B6) and Hst1/Gel-MA group (C1–C6). N = 6 animals per group. The tissues were retrieved at 4 weeks post operation and then subjected to histologic processing and sectioning. The dotted square area is the original defect area (3mm*3mm). Scale bar = 500 μm.
